# A global call for action to tackle skin-related neglected tropical diseases (skin NTDs) through integration: An ambitious step change

**DOI:** 10.1371/journal.pntd.0011357

**Published:** 2023-06-15

**Authors:** Rie R. Yotsu, L. Claire Fuller, Michele E. Murdoch, Wim H. van Brakel, Chandrakant Revankar, Mahoutondji Yves Thierry Barogui, Jose Antonio Ruiz Postigo, Daniel Argaw Dagne, Kingsley Asiedu, Roderick J. Hay

**Affiliations:** 1 Tulane School of Public Health and Tropical Medicine, New Orleans, Louisiana, United States of America; 2 School of Tropical Medicine and Global Health, Nagasaki University, Nagasaki, Japan; 3 Department of Dermatology, National Center for Global Health and Medicine, Tokyo, Japan; 4 International Foundation for Dermatology, London, United Kingdom; 5 Chelsea and Westminster Hospital NHS Foundation Trust, London, United Kingdom; 6 Department of Dermatology, West Herts Teaching Hospitals NHS Trust, Watford General Hospital, Watford, United Kingdom; 7 NLR International, Amsterdam, the Netherlands; 8 Elimination of NTDs (Independent), North Brunswick, New Jersey, United States of America; 9 World Health Organization, Regional Office for Africa, Libreville, Gabon; 10 World Health Organization Headquarters, Geneva, Switzerland; 11 King’s College, London, United Kingdom; Saudi Ministry of Health, SAUDI ARABIA

## Abstract

On 8 June 2022, the World Health Organization (WHO) released pivotal guidance, “Ending the neglect to attain the Sustainable Development Goals: A strategic framework for integrated control and management of skin-related neglected tropical diseases.” Skin-related neglected tropical diseases, or skin NTDs, comprise a group of NTDs that produce signs and symptoms on the skin and include at least 9 diseases or disease groups. Moving away from disease-specific approaches, it is anticipated that synergies will be identified and integrated building on this shared feature, where possible, to achieve a greater health impact. This paper intends to draw attention to the prospects created by this scheme. The framework is a key basis for a proposal produced by WHO dedicated to skin NTD integration and describes the practical opportunities for this evolving strategy. It underlines the wider health benefits that will follow, thus working towards Universal Health Coverage and skin health for all.

## Introduction

On 8 June 2022, the World Health Organization (WHO) published “Ending the neglect to attain the Sustainable Development Goals: A strategic framework for integrated control and management of skin-related neglected tropical diseases” [1]. It is a companion document to “A road map for neglected tropical diseases 2021–2030” by WHO, which sets global targets and milestones to control, eliminate, or eradicate Neglected Tropical Diseases (NTDs), as well as cross-cutting targets aligned with the Sustainable Development Goals [2]. It also follows on from earlier WHO initiatives including the position paper published in 2017, manual for “Recognizing Neglected Tropical Diseases through their changes on the skin,” and the subsequent development of a Skin NTD diagnostic app [3,4]. The initial call was consolidated by bringing together all interested parties and agencies for a wide-ranging meeting held in March 2023 in Geneva [5,6].

Skin-related neglected tropical diseases, or the skin NTDs, comprise a group of NTDs that produce signs and symptoms on the skin creating a common pathway on which to build care strategies. Building on this shared feature, skin NTD integration has been identified as one of the areas with a cross-cutting focus that will facilitate the delivery of effective outcomes outlined in the road map. This is a strategic shift from the conventional disease-specific approaches which led to, at times, duplication of effort and competing access to limited resources. The framework was developed with the aim of identifying and explaining the practical opportunities for integration across the skin NTDs and to enable endemic countries to adapt the recommendations and incorporate them into their national health plans and programmes.

This report describes the essentials of the framework and sets out potential ways to achieve skin NTD integration. Globally, it is known that skin diseases are the 18th leading cause of disability-adjusted life years (DALYs) and fourth leading cause of disease [7]. It is anticipated that this initiative will contribute towards achieving better care for those affected by skin NTDs, while underlining the wider health benefits for all that will follow in particular those with common skin diseases.

### Skin NTDs—The more neglected among the NTDs

NTDs are a diverse group of diseases that are prevalent mainly in low- and middle-income countries (LMICs) and in underserved communities. These diseases have been relatively “neglected” by public health initiatives, research, and drug development. Together, they have a considerable adverse impact on the health and well-being of populations. Twenty diseases and disease groups are listed as NTDs by WHO, and integrated approaches are being promoted to allow for more effective disease control and health improvement by sharing resources and opportunities [2]. Integration in this context refers to NTD-related activities that have an impact on more than one of these conditions at any given time.

At least 9 of the NTDs comprise the group of skin NTDs. Many of these cannot be prevented by mass drug administration (MDA), which is a public health intervention delivered through treating entire populations at risk in selected geographical areas. Rather, they require individual-based diagnosis and treatment, which may be long term and often resource demanding. Lack of capacity as well as training and infrastructure among healthcare workers in the remote settings where skin NTDs are endemic has hindered the delivery of adequate care to those affected [8]. As the impact of these interventions is more difficult to measure, this group of NTDs has been even more neglected than those predominantly targeted by MDA. Furthermore, most skin NTDs may cause chronic disabilities and deformities, often for life, if not diagnosed and treated early. As a result, people affected by skin NTDs may be subjected to considerable stigma and discrimination leading to further isolation and vulnerability to poverty—creating a vicious cycle. Together, all these affect the mental well-being and quality of life, of those affected and that of their family members. While there are, as yet, no formal surveillance systems for some of these diseases, leading to both under- and overreporting, it is estimated that more than 1 billion people are either at risk of, or affected by, one or more of the skin NTDs [2].

### Transition from disease-specific to combined and integrated programmes

There are many reasons for promoting disease-specific programmes that range from concentrating public health focus to simplification of research objectives and public fund raising. However, recognition of the individual aspects of diseases and their expression in populations is but one aspect of human health. The trick here is to identify the similarities and promote integration, while remaining aware of the nature of individual diseases and embedding strategies for their elimination and control within a common framework. Integration does not imply completely abandoning some disease-specific activities or interventions. They are as important and could still be implemented in the context of integrated service delivery.

**Fig 1:**
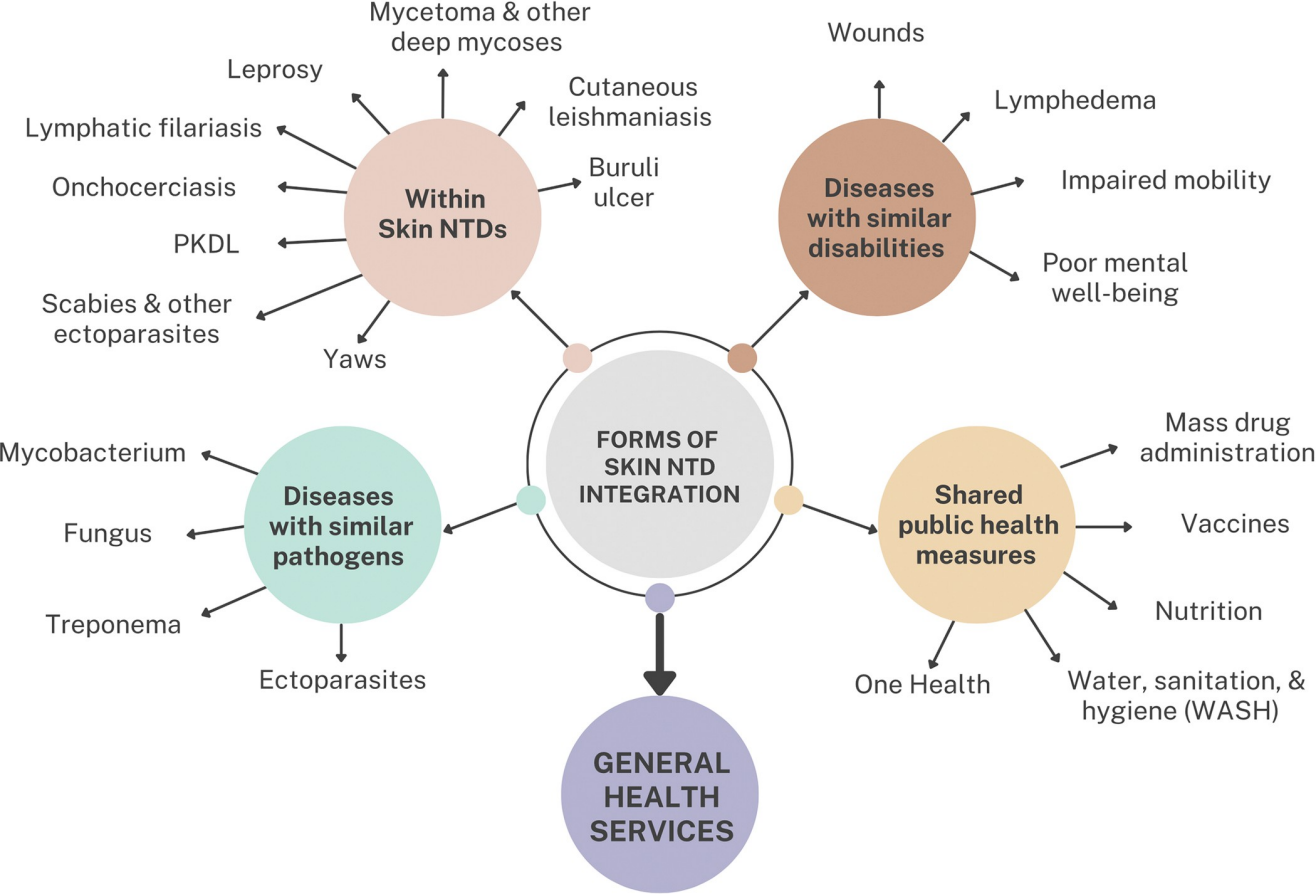
Mapping out potential combined and integrated programmes.

Integration in a broader sense can range from combining of 2 or 3 diseases or programmes to having the disease embedded into the health system. For example, in some countries, leprosy is managed together with tuberculosis as they are both mycobacterial diseases, from the same group of pathogens, sharing many control strategies. In India, the highest burdened country with endemic lymphatic filariasis, the combination of allopathic and traditional healing methods in the management of lymphoedema has provided patients with limb swelling, irrespective of cause, with successful outcomes [9]. These are some forms of integration, by disease or by symptom, respectively. Yet, another form of integration is that diagnoses of skin NTDs are provided as a part of general dermatological services and no specific programmes exist for any individual diseases. Fig 1 maps out these different forms of combined and/or integrated programmes. The term can be used interchangeably depending on the context.

### Skin NTD services—Multidisciplinary medicine and regional adaptation

**Fig 2:**
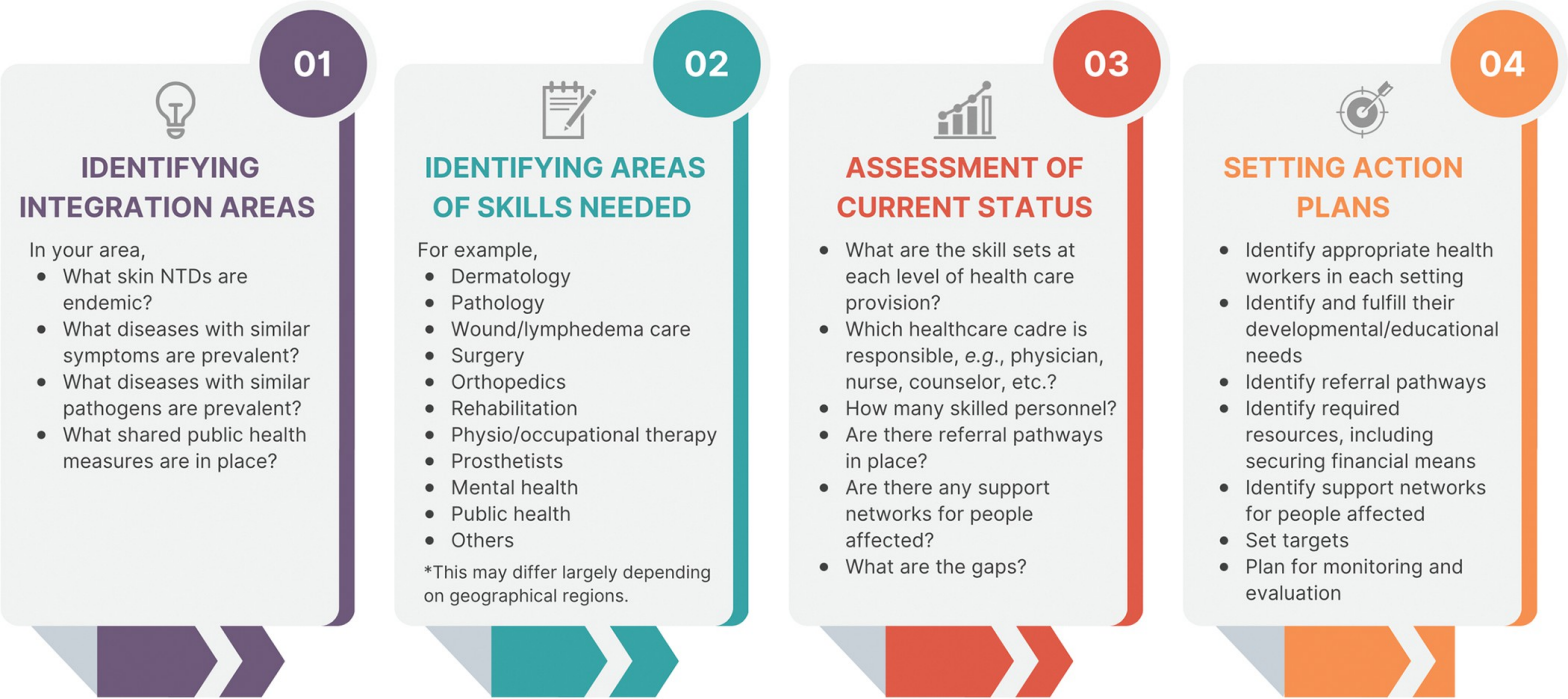
Process for developing action plans for skin NTD integration.

Health services for skin NTDs are very complex. In terms of clinical discipline, dermatology is often regarded to be the key, but dermatology alone cannot address many of the conditions and complications that surrounds the skin NTDs. Surgery, orthopaedics, rehabilitation, pathology, mental health, among many others, are also essential and similarly important—so-called “multidisciplinary medicine.” Understanding this complexity of skin NTD services available to patients is vital for countries planning skin NTD control strategies. The needs will also differ in different parts of the world as areas of skin NTD co-endemicity vary geographically. Mapping out the interplay between community, primary, secondary, and tertiary care services appropriate to the location enables the identification of appropriate health workers in each setting as well as their developmental/educational needs to build an appropriately equipped multidisciplinary network across the endemic country health system (Fig 2). While patients should be referred early for more specialised advice when necessary, including confirmation of diagnosis by laboratory methods, it is also as important that, where possible, local clinics should remain the main providers where patients can continue to receive care close to their homes.

None of the work described here can happen if there is no access to adequate specialist knowledge and skills on the ground. There are still a limited number of qualified dermatologists or specially trained health assistants in LMICs where skin NTDs are endemic, or they are concentrated in very large urban centres, and this service gap needs to be addressed urgently if integrated skin NTD programmes are to succeed [10]. In a similar way, access to skills in nursing, wound care, mental health, and rehabilitation are needed, and health systems need to adapt to embrace this strategy. This will ensure country ownership and plans for sustainability of the programmes put in place.

### People- or person-centred approaches as a key driver to the design of integrated diagnostic and management schemes

Attention to the journey that people affected by skin NTDs follow is a strategic focus of the framework as a key driver to the design of diagnostic and management schemes. Many of those with skin NTDs will have had a long experience with their disease. Understanding the impact of this chronicity in a holistic way, together with exploring potential points of intervention is one of the opportunities offered by this approach. For example, intervening early to prevent disability and stigma and providing support for affected individuals to maintain the capacity to continue education or paid employment are important considerations that limit the burden of the disease on the individual [11].

Furthermore, integrated management schemes can be more effectively delivered by ensuring that people affected by skin NTDs (this includes family members) are placed at the centre of their care; this can be even supervised by peer experts rather than formally trained healthcare workers. For example, in Nepal, attempts are being made to integrate self-care for filarial lymphoedema into existing self-care groups for leprosy at the community health level [12]. In Ethiopia, integrated management for podoconiosis and lymphatic filariasis has shown great potential for combined disease management services, which currently further integrates leprosy and delivers a family-based intervention [13,14]. A similar approach to integrated care could be adopted for management of neuropathic ulcers due to leprosy and diabetes, a neglected chronic disease with growing prevalence in which self-care is largely practiced, together [15].

Many of these projects have one vision in common, which is to achieve cure. The cure from the disease is not just elimination of the infection by medical treatment—it is a state in which the person has fully recovered from the disease, has good mental well-being, and is able to lead a normal life and function in society. Even left with physical disabilities, or impairments, it should not limit this goal, and rehabilitation is aimed at optimising functioning, both in terms of the individual activities of daily living and social and work participation. Nonetheless, impairments should be prevented or mitigated as far as possible. Sometimes, it is not the impairment itself that hinders inclusion and participation of persons affected by skin NTDs, but the stigma and discrimination, which will be discussed later.

### Targeting the population: Social mobilisation, active case detection, and MDA

While individualised care is required for most skin NTDs, opportunities created from activities targeting the population should not be missed to reach a greater impact. Previous examples of integrated skin surveys have shown that it is much more efficient and effective to target several skin NTDs simultaneously, with not only seeing increases in case numbers detected, but also in identifying skin NTD hotspots and in understanding more of their epidemiologies [16]. On the other hand, some important challenges have been highlighted, for example, the large number of common skin diseases that are encountered when using broad case definitions for integrated screening or diagnosis. This burden of common skin disease outweighs that of skin NTDs and can add serious workload for healthcare workers or on to specific activities [16,17]. Moreover, treatment of common skin diseases must be considered in order to promote skin health and improve acceptability of the wider scheme within target communities [16,17].

Social mobilisation, or sensitization, of community members is another area that could be integrated in targeting the population. The basic message to be delivered may be to check one’s skin and access health facilities when abnormal skin signs are detected—which is applicable and can be addressed across all skin NTDs. The format and content of messages used in social mobilisation need to be revisited, and this may lead to a shift from a situation where fear of a specific disease is created to the delivery of broader and more positive messages. Such an approach will ultimately prevent the creation of unnecessary feelings of stigma and discrimination.

To date, MDA programmes have mainly been limited to a group of 5 NTDs (lymphatic filariasis, onchocerciasis, schistosomiasis, soil-transmitted helminthiasis, and trachoma). However, there have been reports on the off-target impact of azithromycin MDA against trachoma in reducing cases of yaws [18,19], and similarly, ivermectin MDA against onchocerciasis and lymphatic filariasis in reducing cases of soil-transmitted helminthiases and scabies [20,21]. As there is this growing evidence of unanticipated benefits, MDA using azithromycin for yaws eradication is currently being carried out as a planned programme in some African and Pacific island countries in order to reach a wider spectrum of disease. Infections like scabies, which are also amenable to ivermectin MDA, could also be included in the future in existing integrated preventive chemotherapy programmes in areas co-endemic for ivermectin-responsive NTDs.

### What tools do we have available for control of skin NTDs?—Sharing of tools

As these diseases are neglected, so are developments in new diagnostic tools and point-of-care tests using modern molecular techniques or in the provision of and training in the use of simple laboratory methods (S1 Table) [22,23]. There have also been few new developments in research and field testing of new treatments (S2 Table).

Direct microscopy is a basic laboratory skill in pathology and clinical medicine. It is also a simple core diagnostic skill for the recognition of many NTDs from cutaneous leishmaniasis to mycetoma and leprosy [24]. However, often, these tests are not performed for various reasons. Sometimes the skills are not available, or even if there is skill and appropriate facilities to perform the tests, the simple expedient sharing of resources across different diseases may not be permitted. These barriers to care should be addressed, and the availability of laboratory tests should be widened and supported to provide the confirmatory diagnosis and appropriate clinical care. Although the foregoing relates to the use of simple laboratory skills, there is an equally urgent need for the development of point-of-care tests for both single and multiplex platforms based on antigen or gene/molecular recognition—to bring diagnostics closer to where patients reside. WHO has recently developed Target Product Profiles (TPPs) to guide the development of priority diagnostic tests for selected NTDs, including for skin NTDs [25,26,27,28]. There is also scope for identifying reference centres for the more complex tests, including assays for drug resistance [29].

While there have been advances in developing new avermectins such as moxidectin for the treatment of skin NTDs like onchocerciasis and scabies [30], the treatment of other skin NTDs including mycetoma and leishmaniasis still relies on old drugs with suboptimal efficacy and whose cost can be prohibitive in the poor communities where the disease is endemic [31,32]. Development of new drugs or repurposing old drugs for these diseases, which are safe, effective, and low cost, is awaited. Clinical trials and systematic reviews of both new and old treatments are also needed. The recent systematic assessments of thermotherapy for cutaneous leishmaniasis is an example of this type of work [33]. Furthermore, addressing the issue of treatment cost, which limits the provision of care in so many places, is a priority [34]. Beyond this, there is also important work to be completed in informing and influencing regulators about the benefits, and also harms, of new treatments.

Recognising disease is a starting point for innovation in this field. While we have generally relied on the human eye, seasoned by practice and experience, as the main method for diagnosis of skin lesions, there are exciting opportunities to extend this to include the use of new algorithms, artificial/augmented intelligence, and visual devices to improve the access to, and accuracy of, diagnosis [35,36]. Likewise, innovation in training methods, whether at distance by telemedicine or through online programmes or more conventional face-to-face teaching, is urgently needed to improve diagnostic capacity of health workers. Assessing the impact of training is crucial and is currently conducted via methods such as pre- and post-course quizzes or number of recipients trained [37]. Using other assessment methods such as field Observed Structural Clinical Examinations (OSCEs) or surrogate measures including reductions in use of ineffective treatments should be explored [38]. Broadening and improving training evaluation techniques to demonstrate lasting change in professional behaviours remains an ongoing need in effective impact assessment.

### Reducing stigma and promoting inclusion into society

It is important to come back to the experience of the person affected and focus on what is important to them. People with skin NTDs have often suffered not just the physical, but also the mental consequences of their disease, especially those whose manifestations are clearly visible to others. They are prone to experiencing stigma and discrimination, resulting in reduced social and work participation, and lowering of their self-esteem [39,40,41]. This aspect of health is often ignored. It is also the stigma associated with these diseases that may prevent patients presenting to healthcare facilities early, leading to development of disabilities and deformities and thus leading to a vicious cycle of further aggravating their experience of stigma as a consequence [42]. It is sometimes also the healthcare providers who treat people with skin NTDs in a demeaning way—which may hinder access to care [43,44].

However, attitudes have started to change over the past 10 years or so, and there have been significant advances in understanding the physical and social impact of skin NTDs. Such changes are often limited to areas and countries where specific action has been undertaken to reduce stigma and discrimination and where those affected have been empowered, for example, through their participation in interventions or through self-organisation [45,46,47]. In addition, initiatives have been promoted where reintegrating those affected into their communities have yielded rich rewards. There are various ways to reduce the impact of stigma and discrimination targeted at different levels. Integration of services for a stigmatised condition into general health services has been shown to have a destigmatising impact in India and Nepal [48,49]. For leprosy, support groups for self-care and livelihoods have helped individuals affected by the disease to develop coping mechanisms and resilience [11,50]. These techniques can be extended to other skin NTDs. Moreover, through integration and sharing of practices, it is hoped that social perceptions of stigma and discrimination against one particular disease will gradually be diluted and mitigated.

Correct information about the disease needs to be delivered to support avoiding the negative images of skin NTDs—messages from experts are often considered credible [51]. This needs to be practiced at every level including for patients themselves and their family members. It is important that messages are contextualised and address local knowledge gaps, misconceptions, cultural and religious beliefs, and fears and are delivered in a form that is readily intelligible for the local population [52]. Knowledge-based interventions are important but often not enough to counter stigma on their own. Contact-based interventions and other approaches that involve persons with lived experience directly have been shown to be effective in multiple intervention studies [50,53,54].

### Impact of skin NTDs on mental well-being of persons affected

A combination of visible, often disturbing and sometimes painful, physical signs, disabling consequences, social stigma, and poverty-related factors have been shown to have an impact on persons affected by skin NTDs and their family members [42,55,56]. The prevalence of symptoms of depression and anxiety is much higher than in the surrounding community and may be as high as 50% in some studies. At the same time, mental health services are often absent or not well developed in areas where skin NTDs are common [56]. The way forward clearly requires a multipronged approach with prevention of mental health problems. This can be achieved through NTD treatment, stigma reduction and disability management, task-shifting of mental health services to lay health workers and even peer supporters through appropriate training using materials such as the WHO mhGAP toolkit and the recently developed Basic Psychological Support for persons affected by NTDs, and establishing referral pathways to professional mental health services [57,58].

### Going beyond skin NTDs

While skin health for all is an ambitious target, the framework demonstrates that it is feasible through careful planning and regional knowledge of prevalent diseases as well as identifying potential suitable interventions to address the most common skin diseases that account for more than 80% of the burden of skin disease in skin NTD endemic areas [17,40,59]. In these settings, around 8 to 10 common dermatological diseases account for most of the communities’ cutaneous disease burden, and focusing on these will address the majority of the local skin health needs. Skin surveys targeting these common skin conditions as well as skin NTDs have been rewarded by a greater acceptability of such programmes by communities [17]. Many share common treatment pathways and, in most cases, low cost medicines are available to address these diseases [17]. This is not to trivialise the challenges, but a firm focus on rationalising procurement and management of drug stocks locally would also help to make significant inroads into addressing this burden of disease. Maintaining clean skin is one of the most effective preventive measures for many skin infections, requiring access to clean water and awareness raising on skin hygiene. Wound care is also important; many Buruli ulcer and leprosy clinics are taking care of ulcers caused by other conditions [60]. Establishment of distance diagnostic and programme support schemes through telemedicine or WhatsApp groups, not limited to skin NTDs, have the potential to reduce the common skin disease burden [61,62,63]. The skin NTD strategic framework recognises the importance of addressing these common diseases as an intrinsic part of delivering a programme that addresses the skin NTDs.

### A way forward

Skin manifestations are the fundamental commonality among the skin NTDs that has promoted integration. However, areas for integration are not limited to interventions related directly to the skin but can be extended to areas including rehabilitation, mental well-being, and reduction of stigma and discrimination. It is expected that innovative integration will happen through successfully utilising available resources and opportunities, which may also go beyond skin NTDs and ensure they are embedded within endemic country health services to ensure sustainability. As we have shown here, professionals, researchers, and public health workers operating within a multidisciplinary environment are needed to help and lead in designing integrated skin NTD programmes. Through the actions taken, persons affected by skin NTDs as well as locally dominant skin diseases should be able to receive high-quality and continued care, while living stigma free with their families and in their communities in order to achieve the goal of *skin health for all*.

## Supporting information

S1 Table Diagnostic methods and tools for skin NTDs. (PDF)

S2 Table Treatment and management of skin NTDs. (PDF)
