# Supplementary material for: A global call for action to tackle skin-related neglected tropical diseases (skin NTDs) through integration: An ambitious step change
Source: PLoS Negl Trop Dis. 2023 Jun 15;17(6):e0011357. doi: 10.1371/journal.pntd.0011357 (PMC10270348; doi:10.1371/journal.pntd.0011357)
Supplement: S1 Table — (PDF) [file pntd.0011357.s001.pdf]

Table 1. Diagnostic methods and tools for skin NTDs

	Pathogen	Rapid diagnostic test	PCR	Microscopy	Culture	Serology	Others
**Buruli ulcer**	*Mycobacterium ulcerans*	X	O	O	O	X	LAMP test, thin layer chromatography, antigen detection assays under development
**Cutaneous / mucocutaneous leishmaniasis**	*Leishmania* species	X	O	O<br>Skin smears	O	X	LAMP test, antigen detection assays under development<br>(Montenegro skin test)
**Mycetoma, chromoblastomycosis, and other deep mycoses**	Fungal or bacterial species	X	O	▵	O	X	X-rays, CT, ultrasonography, etc.
**Leprosy (Hansen's disease)**	*Mycobacterium leprae*	X	O	O	X	▵<br>Anti-PGL-I antibody	Thickened nerves, loss of muscle strength, anesthetic skin lesion
**Lymphatic filariasis**	Microfilaria (*Wuchereria bancrofti*, *Brugia malayi*, etc.)	O	O	O<br>Blood smears	X	▵<br>Anti-filarial antibodies	Ultrasonography
**Onchocerciasis**	Microfilaria (*Onchocerca volvulus)*	X	O	O<br>Skin snips	X	▵<br>Anti-filarial antibodies	Slit-lamp eye exam, ultrasonographic observation of adult worms in nodules or direct observation of adult worms in excised nodule(s), serological and antigen tests under development

**Post kala-azar dermal leishmaniasis (PKDL)**	*Leishmania* species (mainly *L.donovani*)	X	O	△<br>Skin smears	O	X	Mainly clinical diagnosis
**Scabies**	*Sarcoptes scabiei* var. *hominis*	X	△	O	X	X	Dermatoscopy, burrow ink test
**Tungiasis**	*Tunga penetrans* (sand fleas)	X	△	O	X	X	Direct observation of adult fleas and eggs from skin lesion(s),<br>dermatoscopy
**Yaws**	*Treponema pallidum* subsp. *pertenue*	O	O	O	O	PRP, TPHA, FTA-ABS, etc.	Diagnostics for differentiation of *Treponema pallidum* species under development
