# Supplementary material for: A global call for action to tackle skin-related neglected tropical diseases (skin NTDs) through integration: An ambitious step change
Source: PLoS Negl Trop Dis. 2023 Jun 15;17(6):e0011357. doi: 10.1371/journal.pntd.0011357 (PMC10270348; doi:10.1371/journal.pntd.0011357)
Supplement: S2 Table — (PDF) [file pntd.0011357.s002.pdf]

Table 2. Treatment and management of skin NTDs

	Medical treatment	Surgery	Wound or lymphedema management	Self-care	Prevention
**Buruli ulcer**	***Standard:*** Oral rifampicin + clarithromycin for 8 weeks<br>***Other tested regimens:*** Oral rifampicin + either 1 or 2 of [ciprofloxacin, ethambutol, mofloxacin, amikacin, etc.]	Yes	Yes	Yes	Limited, route of transmission unknown (Stay away from contaminated water sources)
**Cutaneous / mucocutaneous leishmaniasis**	Individualized treatment depending on species (no standard)<br><br>Amphotericin B deoxycholate, pentavalent antimonials, fluconazole, ketoconazole, miltefosine (oral), paromomycin ointment, etc.<br><br>***Simple CL*** lesion(s) with low ML-risk: natural healing may occur<br>***Complex CL*** lesion(s) with high-ML risk, severe lesion(s), immunocompromised persons, etc: treat all cases	No	Yes	No	Limited (Avoid sand fly bites)
**Mycetoma**	Antibiotics or antifungals depending on species for long-term	Yes	Yes	Yes	Footwear
**Leprosy (Hansen's disease)**	***Multiple drug therapy (MDT):*** Oral rifampicin + dapsone + clofazimine for 6 to 12 months	Yes	Yes	Yes	Contact tracing and early detection; prophylaxis with one-dose rifampicin in trial
**Lymphatic filariasis**	Oral albendazole ± [diethylocarbamazine (DEC) or ivermectin] at least once yearly for 4-6 years	Yes	Yes	Yes	Avoid mosquito bites, MDAs, vector control, etc.

**Lymphatic filariasis (continued)**	*DEC contraindicated in co-endemic sites with onchocerciasis; ivermectin contraindicated in co-endemic sites with loiasis<br><br>When long-term treatment is possible: Oral diethylocarbamazine (DEC) (1-12 days) ± doxycycline for 4 to 6 weeks				
**Onchocerciasis**	Oral ivermectin at least once yearly for 10-15 years<br><br>*Ivermectin contraindicated in co-endemic sites with loiasis	Yes	No	No	Avoid blackfly bites, MDAs, vector control, etc.
**Post kala-azar dermal leishmaniasis (PKDL)**	Individualized treatment depending on the stage<br><br>Amphotericin B deoxycholate, pentavalent antimonials, miltefosine (oral), etc.	No	No	No	Adherence to treatment for visceral leishmaniasis
**Scabies**	Oral ivermectin, 1-2 doses 1 week apart	No	No	No	Early diagnosis and treatment of contacts, possible MDAs in endemic communities
**Tungiasis**	None (primary treatment: hygienic mechanical removal of fleas), antibiotics if secondary infection is indicated	No	Yes	No	Footwear
**Yaws**	Single oral azithromycin or injectable benzathine penicillin	No	Yes	No	Contact tracing and early detection, possible MDAs in endemic communities
